# ANKLE BRACHIAL INDEX (ABI) AND ACCELEROMETER PATTERNS OF DAILY PHYSICAL ACTIVITY IN HCHS/SOL

**DOI:** 10.1093/geroni/igac059.1304

**Published:** 2022-12-20

**Authors:** Jennifer Schrack, Venus Chiu, Amal Wanigatunga, Daniela Sotres-Alvarez, Shoshana Ballew, Matthew Allison, Robert Kaplan, Kunihiro Matsushita

**Affiliations:** Johns Hopkins Bloomberg School of Public Health, Baltimore, Maryland, United States; Johns Hopkins University, Baltimore, Maryland, United States; Johns Hopkins Bloomberg School of Public Health, Baltimore, Maryland, United States; University of North Carolina, Chapel Hill, North Carolina, United States; Johns Hopkins University, Baltimore, Maryland, United States; University of California San Diego, La Jolla, California, United States; Albert Einstein College of Medicine, Bronx, New York, United States; Johns Hopkins University, Baltimore, Maryland, United States

## Abstract

The benefits of routine engagement in physical activity (PA) on vascular health are well established; lesser known is whether vascular health associates with accelerometry-based daily patterns of PA in mid-to-late life. Using linear regression models adjusted for demographics and comorbidities, we modeled the cross-sectional associations between ankle brachial index (ABI) and PA patterns (diurnal activity and activity fragmentation) in 7,848 HCHS/SOL participants (45.5% men, 56.4±0.2 years). Compared to those with normal ABI (1.0-1.4), those with low ABI (≤0.9) had more fragmented daily activity (B=1.0%, SE=0.4, p=.04); primarily between 12 and 8 PM (B=1.66%, SE=0.55, p<.01). Similarly, those with low ABI had lower daily activity throughout the day (B=-0.09 log-counts, SE=0.05, p<.05) also mainly between 12 and 8 PM (B=-0.16 log-counts, SE=0.55, p<.01). Results suggest a diminished, less favorable PA profile patterns in those with low ABI. Future longitudinal studies should consider whether patterns of PA assist in determining PAD risk.

